# Performance of different clinical trial designs to evaluate treatments during an epidemic

**DOI:** 10.1371/journal.pone.0203387

**Published:** 2018-09-11

**Authors:** Matthias Brueckner, Andrew Titman, Thomas Jaki, Amanda Rojek, Peter Horby

**Affiliations:** 1 Department of Mathematics and Statistics, Lancaster University, Lancaster, United Kingdom; 2 Centre for Tropical Medicine and Global Health, University of Oxford, Oxford, United Kingdom; Universidade Nova de Lisboa Instituto de Higiene e Medicina Tropical, PORTUGAL

## Abstract

In the 2013-2016 west Africa outbreak of Ebola Virus Disease (EVD), most of the planned clinical trials failed to reach a conclusion within the time frame of the epidemic. The performance of clinical trial designs for the evaluation of one or more experimental treatments in the specific context of an ongoing epidemic with changing case fatality rates (CFR) and unpredictable case numbers is unclear. We conduct a comprehensive evaluation of commonly used two- and multi-arm clinical trial designs based on real data, which was recorded during the 2013-16 EVD epidemic in west Africa. The primary endpoint is death within 14 days of hospitalization. The impact of the recruitment start times relative to the time course of the epidemic on the operating characteristics of the clinical trials is analysed. Designs with frequent interim analyses with the possibility of early stopping are shown to outperform designs with only a single analysis not only in terms of average time to conclusion and average sample size, but also in terms of the probability of reaching any conclusion at all. Historic control designs almost always result in substantially inflated false positive rates, when the case fatality rate changes over time. Response-adaptive randomization may be a compromise between the goal of scientific validity and the ethical goal of minimizing the number of patients allocated to ineffective treatments.

## Introduction

In response to the 2013-16 Ebola Virus Disease (EVD) epidemic in west Africa, the international community has called for a strengthening of the research and development response to outbreaks of emerging infectious diseases [1–3]. This enhanced research response is intended to provide an evidence base for the clinical care of patients, including the development of treatments that can improve survival and help contain case numbers [4].

However, how best to evaluate promising treatments during an epidemic is unclear. Phase II, and III clinical trials are required to evaluate drug safety and efficacy (and therefore for licencing), but can only be undertaken when infected patients are available to be enrolled. In previous outbreaks, very few of the planned clinical treatment trials have successfully completed within the time frame of the epidemic. Clinical trials have been either mounted too late to enrol sufficient case numbers, or were unable to reach ambitious recruitment targets [5].

Innovations in the design and conduct of clinical trials may increase the chances of generating reliable evidence. In particular, there is a need to adapt approaches to better meet the practical realities of outbreak epidemiology; that there may only be a short time-course in which to conduct research, that outbreaks may occur over expansive geographical regions, and that case numbers can be unpredictable. Whether the frequently used trial designs, including randomized controlled trials, are the most appropriate choice under these circumstances has been debated [6–10] and further quantitative investigation of the most appropriate trial designs to maximise the likelihood that a planned trial will reach a valid statistical outcome is required.

In this paper we evaluate the performance of commonly used trial designs when applied to an epidemic scenario, as encountered during the 2013-16 EVD epidemic in West Africa. In order to determine their validity and feasibility, the trial designs are compared on the basis of their expected sample size, time to conclusion, false negative probability (risk an effective treatment is declared ineffective), false positive probability (risk an ineffective treatment is declared effective), probability that the best treatment is identified, and the average number of patients assigned to the standard of care arm.

## Data

To inform the simulation model, we analysed a publicly available World Health Organization (WHO) database [11] of individual patient data that included the age, gender, case classification, hopitalisation status, and dates of symptom onset, hospital admission, and outcome (dead or alive) of all cases reported officically to the WHO during the EVD outbreak in Guinea, Liberia and Sierra Leone (last updated on 28 September 2015). The cleaned dataset includes a total of 33338 suspected cases of EVD of which 13506 case were confirmed and hospitalized.

**Fig 1:**
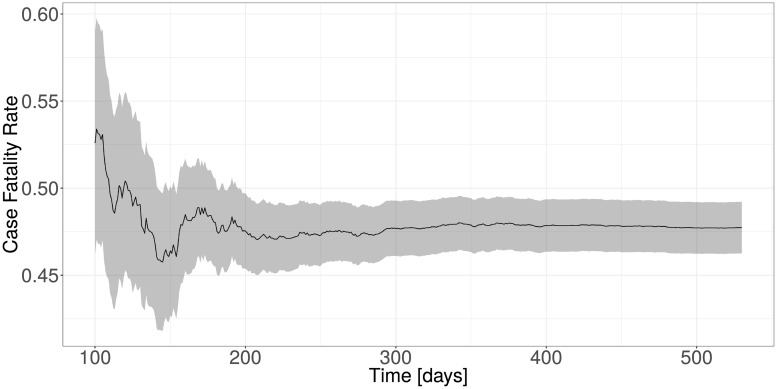
Estimated case fatality rate over time with confidence intervals of all confirmed and hospitalized cases.

A total of 9114 out of 13506 records had missing hospitalization times (6394), missing outcome date (7867) or missing outcome (6781). The problem of missing data is less critical here, since we do not draw any conclusions directly from the observed data, but instead only use this data to inform our simulation model. The objective is to be able to simulate data that has similar characteristics to the data observed in the 2013-16 EVD epidemic in west Africa. We therefore have removed all of the 9114 cases where at least one of the three variables was missing. The simulation model was built on the remaining 13506 − 9114 = 4392 complete cases. The estimated CFR over time for these cases is shown in Fig 1.

## Methods

**Fig 2:**
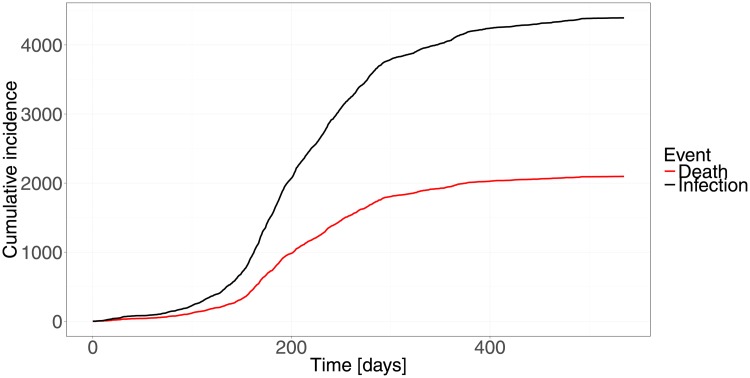
Cumulative incidence of confirmed and hospitalized infections and deaths as a function of days since first case.

The trial program is triggered when a fixed number of *n*_0_ = 10 confirmed and hospitalized cases have been reported. This leads to a trigger time of 11 days after the report of the first confirmed and hospitalized case. Recruitment of the trial is then started with a delay caused by the time it takes to setup the trial. In order to explore the effect of different setup times relative to the observed time course of the epidemic (Fig 2), the delay is chosen such that recruitment starts 100, 200, 300 and 400 days after the start of the epidemic. The total time to conclusion of the trial (including any analysis delays) is design and scenario specific.

### Data generation

Data generation in our simulations is based on the real EVD dataset described above. In our simulation only hospitalized patients with confirmed Ebola virus infection are recruited. The recruitment times in the simulation are randomly sampled from the observed hospitalization times in the original EVD dataset. The number of patients that can be recruited per day is limited to 10. Each recruited patient is randomized to either the control arm or any of the *J* ≥ 1 experimental treatment arms. The details of the randomization procedure are design specific and are given in the clinical trial design descriptions below.

One could be tempted to build a univariate survival model for time to death and to consider recovered patients as censored observations. However, such an analysis would not be valid since the basic “independent censoring” assumption would be violated. Patients that survive EVD tend to be in better health overall than those dying, which means their unobserved death times also tend to be larger. Time to recovery and time to death need to be treated as competing risks [12], since observing one precludes the observation of the other (at least within the scope of our analysis, since a recovered patient is discharged from the treatment center and not followed-up until the eventual death).

**Fig 3:**
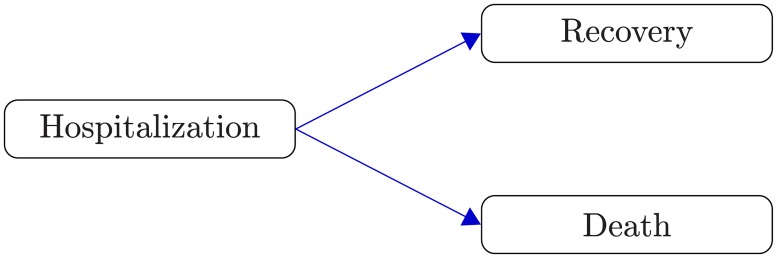
Competing risks. The two possible outcomes for a hospitalized patient are recovery from EVD or death.

For each patient a failure time and its cause (recovery or death) is generated from a competing risks model (Fig 3) with distributions determined by the two conditional cause-specific hazards for recovery *h*_*R*_(*t*|*Z*, *E*) and death *h*_*D*_(*t*|*Z*, *E*), respectively, given treatment arm *Z* and recruitment time *E*. For both cause-specific hazards a proportional hazards model with recruitment time specific hazard ratios is assumed in each treatment arm. For *j* = 0, …, *J*

$$\begin{array}{c} \begin{array}{cc} h_{R}\left(t|Z=j,E=e\right) & =h_{R0}\left(t\right)e^{\beta_{R}\left(j,e\right)}\\ h_{D}\left(t|Z=j,E=e\right) & =h_{D0}\left(t\right)e^{\beta_{D}\left(j,e\right)}, \end{array} \end{array}$$

where *β*_*R*_(0, *e*) = *β*_*D*_(0, *e*) = 0 for all *e*.

The baseline cause-specific hazards *h*_*R*0_ and *h*_*D*0_ are obtained by fitting a flexible parametric spline model [13] to the observed data using the R package flexsurv [14]. Details of this model are described in S2 Appendix. No drop-out or loss to follow-up is considered in the simulations. Censoring only occurs when the follow-up of patients, who have been recruited less than 14 days before an (interim) analysis and have not yet recovered or died, is truncated at the time of the analysis (administrative censoring). Once failure and censoring times are known, the clinical endpoint can be determined.

### Endpoints

The choice of a clinical endpoint is an important aspect in the design of clinical trials. In the context of an emerging epidemic with high mortality a balance must be found such that the endpoint can be observed quickly but is nevertheless clinically meaningful. We select survival to Day 14 post-randomization as the primary endpoint for our evaluation as it seems to be the most frequently used endpoint in EVD trial proposals [8, 15, 16]. This endpoint is 1 for a patient who is still alive at Day 14 after randomization and 0 otherwise. The Day 14 case fatality rate *p*_*j*_ in Arm *j* is estimated by ${\hat{p}}_{j}=\text{C}\hat{\text{I}}{\text{F}}_{j}\left(14\right)$, where $\text{C}\hat{\text{I}}{\text{F}}_{j}$ is the estimate of the cumulative incidence function (CIF) of death in Arm *j* (see S1 Appendix). The advantage of estimating the case fatality rate using the cumulative incidence function is that we could consider the case fatality rate at different times from the same underlying survival model (e.g. at Day 14 and Day 28), and can handle potential administrative right-censoring caused by (interim) analyses.

### Evaluation criteria

In the context of an ongoing epidemic of a severe disease, the performance of a trial design must be evaluated taking into account the large uncertainty about reaching the recruitment target, the goal of quickly finding the most effective treatment, and the ethical problem of randomizing patients to standard of care for a disease with high mortality. The following evaluation criteria will be considered in our comparison of the trial designs:

1. False positive rate (Type I error):
  A false positive decision, i.e. an ineffective experimental treatment is declared superior to the standard of care when it is not.
2. False negative rate (Type II error):
  The probability of declaring a superior treatment ineffective.
3. Average time to a conclusion:
  This depends on the total number of patients recruited and any analysis delays. Designs that allow for early stopping at an interim analysis will on average reach a conclusion much faster than fixed sample size designs. More complex designs might require more time to analyse the data.
4. Average sample size:
  The average sample size is closely related to time to conclusion. Sequential trials with possible early stopping at an interim analysis will recruit fewer patients on average and therefore reach a conclusion faster.
5. Best treatment identified:
  When several experimental treatments (with at least one effective treatment among them) are tested in parallel, the probability of identifying the best treatment instead of only identifying any effective treatment becomes a relevant operating characteristic. In the two-arm case the probability of finding the best treatment (given that the experimental treatment is superior) is simply the statistical power of the trial.
6. Average number of patients treated with ineffective treatment:
  A trial design which minimizes the number of patients allocated to an ineffective treatment arm might be preferable from an ethical perspective and also increase the willingness of eligible patients to participate.

### Clinical trial designs

In this section we give a short description of the 6 two-arm and 3 multi-arm candidate designs that we consider in our evaluation. Commonly used frequentist and Bayesian designs are considered.

**Fig 4:**
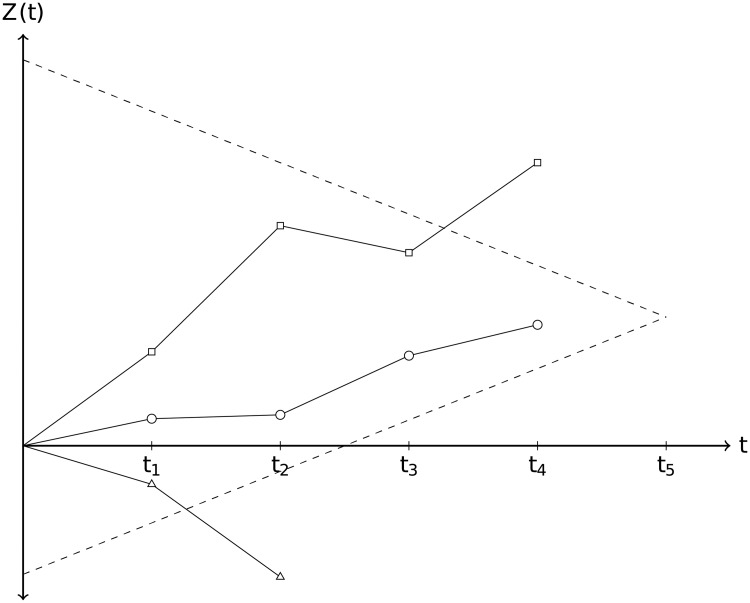
Illustration of a frequentist multi-arm multi-stage design with 3 treatment arms and 5 planned (interim) analyses. Standardized test statistic *Z*(*t*) as a function of time *t* for 3 different treatment arms together with upper (efficacy) and lower (futility) stopping boundaries. In this example the trial would stop at the fourth interim analysis with one treatment crossing the upper boundary (square). One treatment (triangle) would be dropped at the second interim analysis, because it crosses the lower boundary. The third treatment (circle) would be declared ineffective at the time the trial stops.

In general a multi-arm multi-stage starts with a number of experimental treatment arms and a common control arm (standard of care) to which all treatments are compared as illustrated in Fig 4. At each interim analysis a decision for each treatment arm is made to either drop the arm for lack of benefit (stopping for futility), declare superiority over the standard of care (stopping for efficacy), or continue with the next stage. The trial is stopped when either all treatments have been dropped, or at least one treatment is found to be superior to the standard of care or the final analysis is reached.

Design parameters for the two- and multi-arm frequentist designs are chosen such that a one-sided type I error of 2.5% is achieved under the null hypothesis of no randomized treatment effect and a power of 90% for an alternative hypothesis of case fatality rate *p* = 0.4 in the control arm vs. *p* = 0.2 in the experimental treatment arm(s) (without binding futility stopping).

The Bayesian designs offer no formal control of frequentist operating characteristics, but the stopping criteria are tuned such that a false positive rate of 2.5% is achieved for each treatment/control comparison when treatment and control have the same CFR. Using uniform priors for the Bayesian designs results in similar operating characteristics as the frequentist designs.

In frequentist designs the final efficacy boundary is typically set equal to the final futility boundary. The futility boundaries are usually non-binding, i.e. even when the futility boundary is crossed it may be decided to continue the trial (without inflating the type I error) or, at the final analysis, to conclude that the evidence is not conclusive in either direction and more data is needed to declare superiority or futility.

A general flowchart that applies to all designs described in this section is described in Fig A in S3 Appendix.

#### Multi-arm designs

In multi-arm studies *J* ≥ 1 experimental treatments are compared to a common concurrent standard of care control group. Up to 5 equally spaced analyses (including the final analysis) are performed. Efficacy and futility criteria for early stopping are evaluated at each analysis. The trial stops if all experimental treatments have been stopped for futility, or if any of the experimental treatments has been found to be superior. The trial concludes without decision if the maximum sample size or the maximum number of interim analyses has been reached with at least one experimental treatment still active or if the target sample size for the next analysis cannot be reached, because of a lack of eligible patients. Each experimental treatment is compared only to the standard of care arm. No direct comparisons between experimental treatment arms are performed. If an experimental treatment meets the futility criterion it is dropped and recruitment continues only in the remaining treatment arms. The control arm cannot be dropped.

The stopping criteria for the frequentist designs are formulated as thresholds for the standardized test statistic

$$\begin{array}{c} Z_{j}=\frac{{\hat{p}}_{0}-{\hat{p}}_{j}}{\sqrt{{\hat{\sigma }}_{0}^{2}+{\hat{\sigma }}_{j}^{2}}}, \end{array}$$ (1)

where $\left({\hat{p}}_{0},{\hat{\sigma }}_{0}^{2}\right)$ and $\left({\hat{p}}_{j},{\hat{\sigma }}_{j}^{2}\right)$ are the estimated CFR at Day 14 and estimated variance in the control and experimental treatment arm *j*, respectively. It can be shown that this test statistic is asymptotically standard normally distributed.

The Bayesian designs are based on thresholds for the posterior probabilities *P*(*p*_*j*_ < *p*_0_|Data) of an experimental treatment arm having a smaller CFR at Day 14 than the control arm.

All designs aim for a familywise type I error of 2.5% under the null hypothesis of no treatment effect in any of the treatment arms and a power of 90% under the alternative hypothesis of 40% mortality in the control arm vs. 20% mortality in all experimental treatment arms.

In the case of *J* = 4 experimental arms and one control arm a total maximum sample size of *n*_*max*_ = 550 is needed to reach a power of 90%. This results in 22 patients per arm being recruited between any two of the 5 analyses. The sample size is not reallocated when an arm is dropped, i.e. the number of patients allocated to a specific arm does not increase as result of one or more arms being dropped.

1. Multi-arm multi-stage design (MAMS):
  The frequentist MAMS design is based on [17] and stopping boundaries are calculated using the method of [18] based on the same error spending functions as for the two-arm group-sequential design (GSD).
  These boundaries are non-binding, i.e. even if a treatment arm crosses the futility boundary it can still be decided to not drop this arm without inflating the type I error. The values are listed in Table A in S1 Table.
2. Bayesian MAMS with complete randomization (Bayes MAMS):
  We assume the number of observed deaths by Day 14 follows a binomial distribution with unknown case fatality rate *p*_*j*_ in Arm *j*. With a conjugate *Beta*(1, 1) prior, i.e. uniform prior on [0, 1] for each *p*_*j*_, the posterior distribution in Arm *j* is *Beta*(1 + *y*_*j*_, 1 + *n*_*j*_ − *y*_*j*_), where *y*_*j*_ is the number of observed deaths by Day 14 and *n*_*j*_ is the sample size in arm *j* (*j* = 0, 1, …). Superiority and futility criteria are checked for each experimental treatment arm at every analysis and are based on the posterior probabilities of efficacy. The trial is stopped for efficacy, if the posterior probability of superiority of any of the experimental treatments over standard of care exceeds 99%, i.e. *P*(*p*_*j*_ < *p*_0_|Data) > 0.99. An experimental treatment arm is dropped if the posterior probability that the treatment is at least 10% better than the standard of care is less than 10%, i.e. *P*(*p*_*j*_ < *p*_0_ + 0.1|Data)<0.1. The trial is stopped for futility if all experimental treatment arms have been dropped. Superiority and futility thresholds are selected such that the false positive rate is controlled for each treatment-control comparison.
3. Bayesian MAMS with response-adaptive randomization (Bayes RAR MAMS):
  Same as the Bayesian MAMS design, but with response-adaptive randomization, i.e. at every interim analysis the allocation probabilities are recalculated given the observed data, in order to minimize the number of patients allocated to the less effective treatment arm. The trial starts with a balanced allocation among all arms until the first interim analysis to ensure that a minimum number of patients is allocated to each arm. We follow the procedure described in [19] to update the allocation probability at every interim analysis. The posterior probability that treatment *j* is better than every other treatment is *θ*_*j*_ = *P*(*p*_*j*_ < *p*_*k*_∀*k* ≠ *j*|Data) *j* = 0, 1, …, *J*. The new allocation probability for treatment *j* is then proportional to
  $$\begin{array}{c} q_{j}\propto \sqrt{\frac{\theta_{j}V\left(p_{j}\right)}{n_{j}+1}}, \end{array}$$ (2)
  where *V*(*p*_*j*_) is the estimated variance of the case fatality rate and *n*_*j*_ is the current sample size for treatment *j*. The stopping boundaries are the same as for the Bayes MAMS design, i.e. the calculation of the boundaries ignores that the randomization procedure is response-adaptive.

#### Two-arm designs

The two-arm designs are special cases of the multi-arm designs where only one experimental treatment arm is compared to standard of care historic or concurrent control arm.

For the two-arm single stage designs no early stopping is possible and no adjustments for multiplicity are necessary. These designs serve as benchmark for the multi-stage designs that allow for early stopping. A total (i.e. all arms) sample size of 212 is required to achieve 90% power to detect an absolute 20% reduction in the case fatality rate from 40% in the control group to 20% in the experimental treatment arm. For the sequential two-arm designs the total maximum sample size is set to 225 and a maximum of 45 patients is recruited between any two interim analyses.

1. Two-arm single-stage randomized controlled clinical trial (TACC):
  Special case of the MAMS design with one experimental treatment (*J* = 1) and one analysis. The null hypothesis of no treatment effect is rejected if *Z*_1_ > *z*_*α*_, where *z*_*α*_ is the 1 − *α* quantile of the standard normal distribution with *α* = 0.025 in order to achieve a one-sided type I error of 2.5%.
2. Two-arm single-stage historically controlled trial (TAHC):
  All recruited patients are allocated to the experimental treatment arm. The control group is formed by all historic confirmed and hospitalized cases recorded before the start of trial. 105 patients are recruited to the experimental treatment arm (50% of the sample size of the randomized two-arm design). Testing of the null hypothesis is done in exactly the same way as for the designs with concurrent controls. In a disease with very high mortality on standard of care, this design avoids the ethical problem of allocating patients to standard of care when an experimental treatment is available, which is believed to be more effective (otherwise the trial would not be conducted). This design can achieve higher power due to the potentially much larger control group (depending on the trial start). For very early starts, the historic control group may be relatively small however.
3. Group-sequential two-arm RCT (GSD) [20]:
  Special case of the MAMS design with one experimental treatment (*J* = 1). This is a standard one-sided two-arm group-sequential RCT with up to 5 equally spaced interim analysis. Efficacy and futility boundaries are calculated using the R package gsDesign [21] using the default Hwang-Shi-DeCani error spending functions. The values are listed in Table A in S1 Table. The final futility boundary is equal to the final efficacy boundary.

The Bayesian two-arm designs are all special cases of their multi-arm counterparts with one experimental treatment arm (*J* = 1):

- Bayesian two-arm single-stage RCT (Bayes TACC) [22]
- Bayesian Group-sequential two-arm RCT with complete randomization (Bayes GSD)
- Bayesian Group-sequential two-arm RCT with response-adaptive randomization (Bayes RAR GSD)

### Simulation scenarios

We explore the operating characteristics of the trial designs in various scenarios with one or more experimental treatments, which are compared to a common control (standard of care). In order to explore the effect of an improving standard of care during an ongoing epidemic on the operating characteristics of the trial designs, we also consider scenarios where the case fatality rates are decreasing over time, as a result of a linear decrease of the log-hazard ratios of the two cause-specific hazard rates by about 10% over the first half of the epidemic.

We also explore the effect of starting recruitment at different times during the time course of the epidemic. The first cases of West Africa EVD epidemic were reported in March 2014 in Liberia. Studies for evaluating treatments which started enrollment during the epidemic include the Ebola Tx trial [8] (start February 2015) and the PREVAIL II trial [23] (start March 2015). This would roughly correspond to the enrollment start times of 300 and 400 days after the first confirmed and hospitalized case in our simulations. A part of our motivation to evaluate trial designs before an outbreak was the aim of reducing this delay. Therefore we also considered simulation scenarios where enrollment starts only 100 or 200 days after the first confirmed and hospitalized case.

#### Two-arm scenarios

1. No treatment effects. Case fatality rates (CFR) for control and experimental treatment are constant and equal to 0.4
2. No treatment effects, but a linear decrease of the log-hazard ratio in both arms across the first half of the epidemic, resulting in decrease of the case fatality rates from 0.4 at *t* = 0 to about 0.3 at *t* = 250.
3. Experimental treatment (*p*_1_ = 0.2) superior to standard of care (*p*_0_ = 0.4)
4. Experimental treatment (*p*_1_ = 0.2) superior to standard of care, whose mortality decreases from *p*_0_ = 0.4 at *t* = 0 to *p*_0_ = 0.3 at *t* = 250. The decrease in the difference of case fatality rates over time, will result in less power to detect this difference at the same sample size as in Scenario 3.
5. Case fatality rates decreasing in both treatment arms. Experimental treatment (*p*_1_ = 0.2 at *t* = 0 decreasing to *p*_1_ = 0.1 at *t* = 250) superior to standard of care (*p*_0_ = 0.4 at *t* = 0 decreasing to *p*_0_ = 0.3 at *t* = 250).

#### Multi-arm scenarios

The multi-arm scenarios with four experimental treatment arms and 1 common control group are similar to the two-arm scenarios.

- No case fatality rate differences with constant case fatality rates 0.4 in all arms
- No case fatality rate differences, but decreasing case fatality rate from 0.4 at *t* = 0 to 0.3 at *t* = 250 in all arms.
- All experimental treatments (*p*_*j*_ = 0.2, *j* = 1, …, 4) superior to standard of care (*p*_0_ = 0.4)
- Decreasing case fatality rates in all arms, all experimental treatments superior to standard of care (Table 1). Treatment 3 and 4 are the most effective treatments at all times.

**Table 1:** Case fatality rates at the beginning of the epidemic and after 250 days in each arm.

Time [days]	Case fatality rates				
	*p*_0_	*p*_1_	*p*_2_	*p*_3_	*p*_4_
0	0.40	0.34	0.27	0.20	0.20
250	0.30	0.23	0.15	0.10	0.10

## Results

We report the results of 100000 simulations of each of the 9 scenarios with 4 different recruitment starting times (100, 200, 300 and 400 days after the first hospitalized confirmed EVD case). All simulations are done in R [24, 25].

### Two-arm scenarios

**Fig 5:**
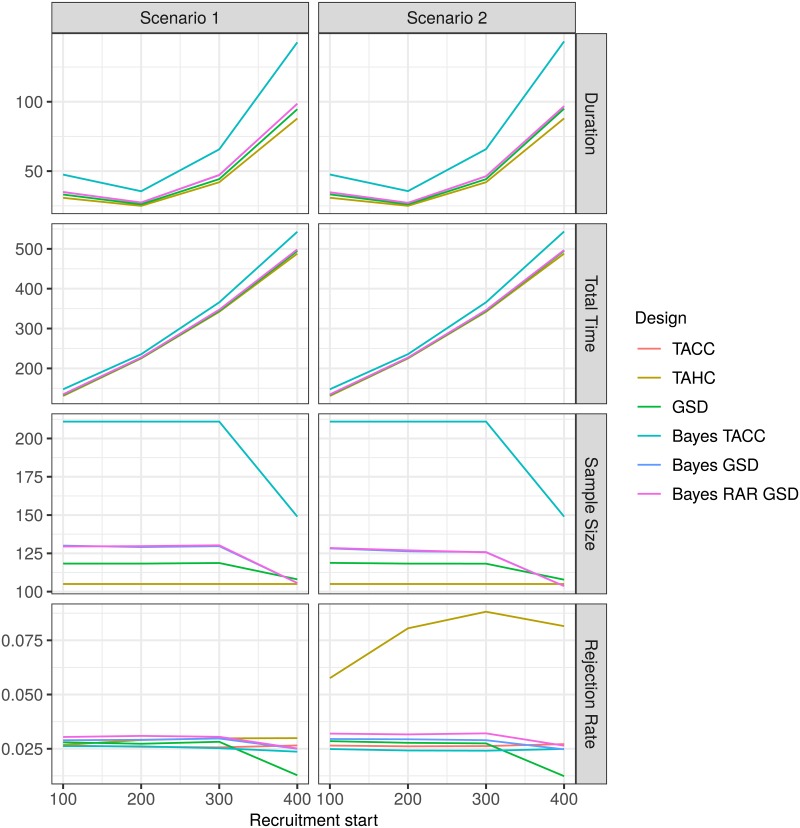
Average duration, average total time to conclusion, average total sample size and rejection rate for each design for the two-arm scenarios 1 and 2 as a function of recruitment start time. For the historic control design (TAHC) only the average number of patients in the experimental treatment arm is shown.

**Fig 6:**
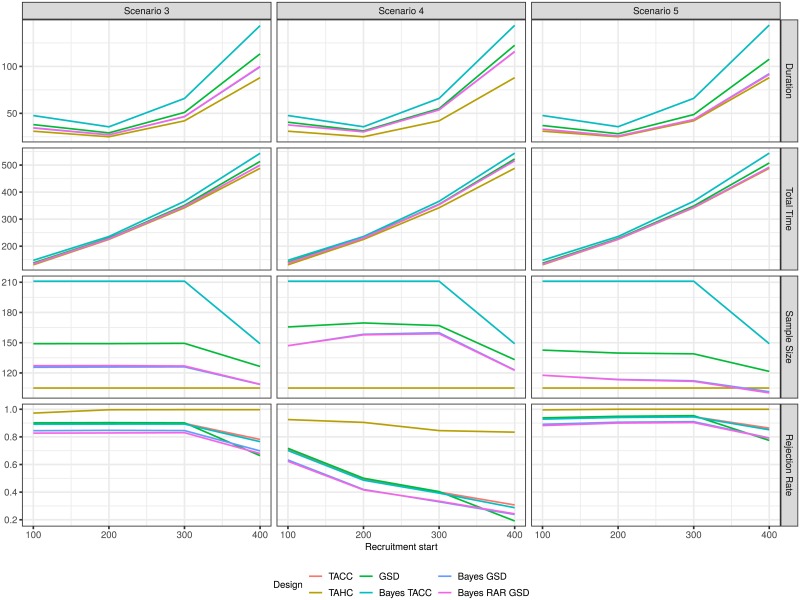
Average duration, average total time to conclusion, average total sample size and rejection rate for each design for the two-arm scenarios 3, 4 and 5 as a function of recruitment start time. For the historic control design (TAHC) only the average number of patients in the experimental treatment arm is shown.

The average duration of the trials first decreases and then increases substantially as recruitment starts later in the time course of the epidemic as a result of a slow down in the rate of new infections (Figs 5 and 6). Starting recruitment during the peak of the epidemic when the number of new infections per day is the highest seems to be optimal in terms of trial duration. However, a trial which is started earlier and runs slightly longer will still conclude earlier than a trial which is started later but runs shorter. The total time it takes to reach a conclusion (measured from the start of the epidemic) increases for all designs with increasing recruitment start time. Note that in the subfigures for average duration, average total time and average total sample size in Fig 5 the line for the frequentist single stage two-arm design (TACC) is completely obscured by the Bayesian single stage two arm design (Bayes TACC).

**Fig 7:**
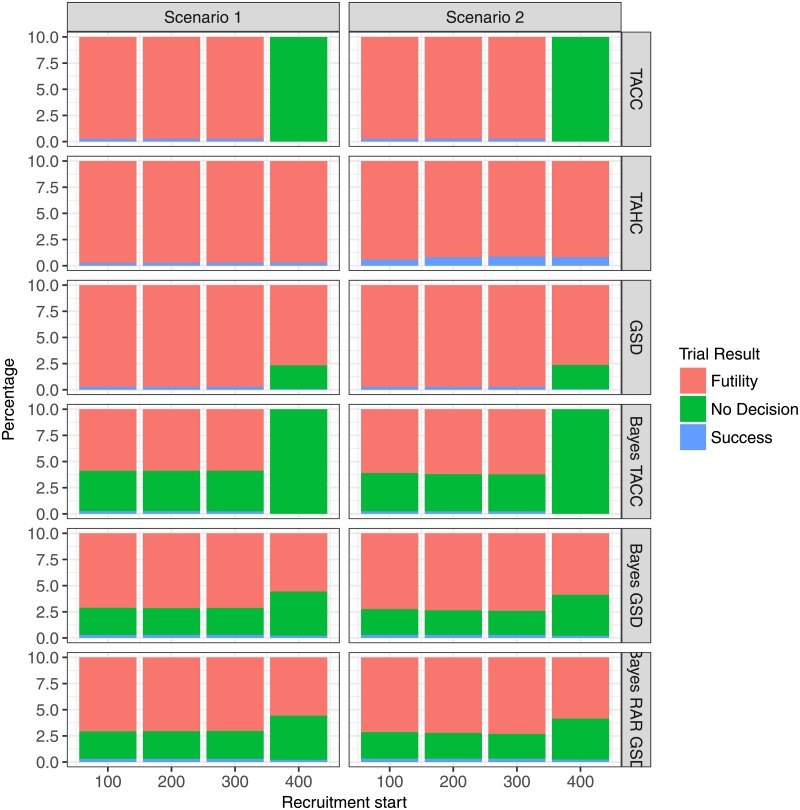
Percentage of trial results for each design for the two-arm scenarios 1 and 2 as function of recruitment start time.

**Fig 8:**
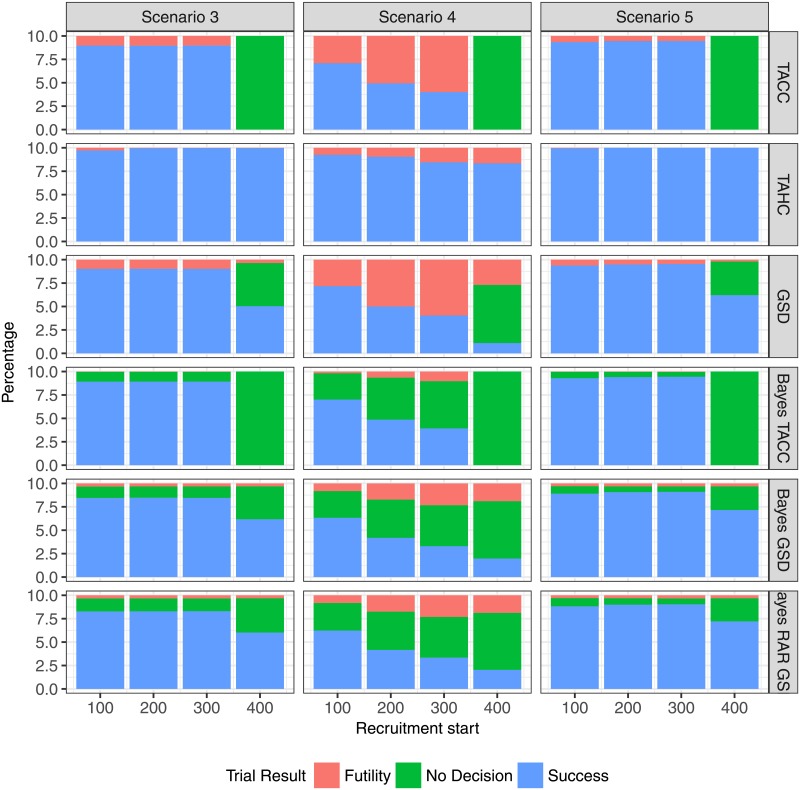
Percentage of trial results for each design for the two-arm scenarios 3, 4 and 5 as function of recruitment start time.

If recruitment begins very late (after 400 days) the planned sample size cannot be reached (Figs 5 and 6). In this case, the single stage designs (TACC and Bayes TACC) conclude without decision in every simulation run (Figs 7 and 8). The historic control design (TAHC) can still reach its recruitment target, since it requires only 50% of the patients to be recruited. It therefore maintains its power whereas all other designs suffer from a substantial decrease in power, because of the smaller sample size. However, the historic control design has a substantially inflated type I error in Scenario 2 (up to 3 times the nominal level), since it compares a group of older controls with a higher case fatality rate to newer patients on the experimental treatment arm. Type I error inflation is expected for the historic control design, and to a lesser degree for response-adaptive randomization design. The type I error of the complete randomization designs is not affected by a simultaneous decrease in mortality in both arms. The sequential designs (GSD, Bayes GSD, Bayes RAR GSD) consistently outperform the single stage designs in terms of average duration and average sample size. The Bayesian sequential designs tend to stop earlier in the scenarios with a non-zero treatment effect than the frequentist group-sequential design. This results in a smaller average sample size.

### Multi-arm scenarios

**Fig 9:**
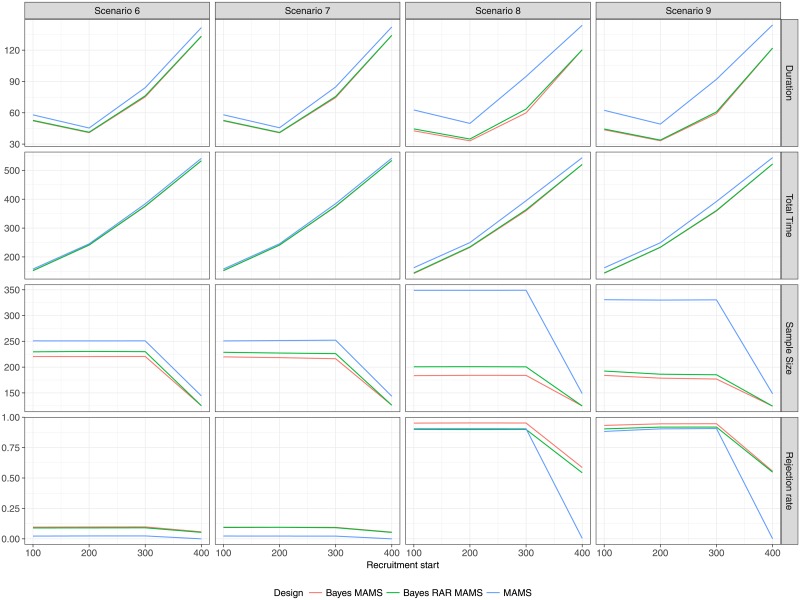
Average duration, average total time to conclusion, average total sample size, rejection rate (of *H*_0_) for each design as function of recruitment start time in the multi-arm scenarios 6-9.

**Fig 10:**
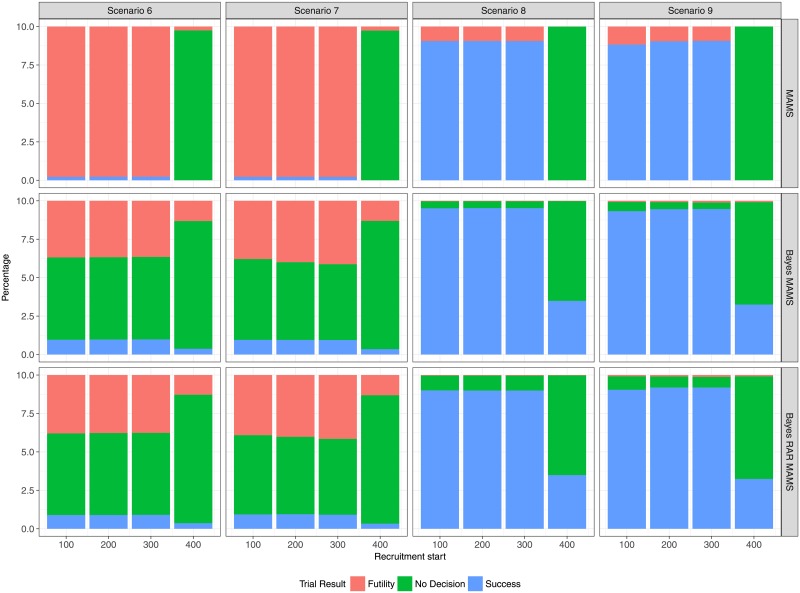
Percentage of trial results for each design as function of recruitment start time in the multi-arm scenarios 6-9.

The results of the multi-arm scenarios (Figs 9 and 10) correspond to the results of the two-arm scenarios. There is almost no difference between the two Bayesian designs with complete and response-adaptive randomization, respectively. However, the differences in terms of average sample size and average duration between the Bayesian and frequentist designs that was seen in the two-arm case is much more pronounced in the multi-arm case. In the two-arm case the largest difference in average sample size is in Scenario 5 with recruitment start after 300 days (139 for GSD vs. 112 for Bayes GSD/RAR). This increase of approximately 24% in sample size results in a difference in average duration of about 5 days (49 days for GSD vs. 44 days for Bayes GSD/RAR). In the multi-arm case the largest difference in average sample size is in Scenario 8 also with a recruitment start after 300 days (349 for MAMS vs 201 for Bayes MAMS and 184 for Bayes RAR MAMS), an increase of 74% and 90%, respectively. The difference in duration is about 30 days (95 days for MAMS vs. 64 and 60 days for Bayes MAMS and Bayes MAMS RAR, respectively). Even with the larger sample size the overall power to detect at least one effective treatment is smaller for the MAMS design, since the Bayesian designs only control the type I error for each treatment-control pair, which results in an almost 4-fold inflation of the overall type I error.

In Scenario 9, experimental treatment arms 3 and 4 are the most effective. Table 2 shows the probability of correctly identifying either one or both of those two treatments as superior to standard of care. The Bayesian MAMS design with complete randomization has a slight advantage over the Bayesian RAR and frequentist MAMS designs. The two Bayesian designs at least have a probability of about 40% of correctly identifying an effective treatment even for a very late start of the trial, whereas the frequentist MAMS design ends with no decision in almost every case, because it fails to reach its recruitment target. From Table 3 it can be seen that the response-adaptive design indeed allocates fewer patients to the control group in Scenarios 8 and 9 than any other design.

**Table 2:** Probability of identifying experimental treatment 3 or 4 as effective in scenario 9.

Prob. best treatments identified (%)			
Start	Bayes MAMS	Bayes RAR MAMS	MAMS
100	65.40	61.30	64.10
200	68.45	63.79	65.90
300	68.95	63.89	66.93
400	39.90	38.37	0.07

**Table 3:** Average number of patients allocated to control group (Arm 0) for scenarios 8 and 9 and different recruitment start times.

Average number of patients in Arm 0				
Scenario	Start	Bayes MAMS	Bayes RAR MAMS	MAMS
8	100	28	23	44
	200	28	23	44
	300	28	23	44
	400	21	19	23
9	100	30	24	45
	200	29	24	44
	300	28	23	44
	400	22	19	23

We also investigated different treatment effect sizes in simulations not reported here and the conclusions remained the same, since all designs were affected in a similar way.

## Discussion

We demonstrate how integrating outbreak epidemiology into the selection process for a clinical trial design can help streamline clinical trials during outbreaks.

In general there were no substantial differences between the single stage Bayesian and frequentist designs when using non-informative priors, as would be expected. However, in the sequential case the Bayesian designs required a smaller sample size and reached a conclusion faster on average than their frequentist counterparts. These differences were substantial in the multi-arm case. The implications for practice are that a faster conclusion may enable role-out of an efficacious treatment into larger confirmatory studies before the outbreak wanes, or allow a non-efficacious approach to be abandoned for trials of other possible treatments.

An important difference between the designs as implemented here is that the Bayesian designs may conclude the trial without decision at the final analysis, whereas the frequentist designs force a decision at the final analysis by setting the futility boundary to be equal to the efficacy boundary. In our simulations a “no decision” result is only possible if the final analysis cannot be reached, because of a lack of new patients. However, the futility boundaries are non-binding and in practice it may still be concluded that further data is required even after the boundary has been crossed. When the treatment effect is smaller than anticipated, e.g. when improving standard of care in both arms reduces the difference in treatment effect between treatment and control, the designs will be underpowered. In terms of practical approaches, not forcing a decision for futility or superiority might be the most appropriate action in such a situation.

Interim analyses are beneficial, leading to smaller and faster trials on average (at the price of a small power loss at the same maximum sample size). A trial with interim analysis may be successful, by stopping early for efficacy, even when the planned maximum sample size could not be reached, due to a waning of the epidemic. Even more important are the ethical implications of early stopping for futility, reducing the number of patients exposed to an ineffective treatment.

Designs using historical controls are problematic when the treatment effects change over time, e.g. due to improvement in the standard of care, although they may have higher power due to a potentially much larger control arm. Furthermore, when the trial is started very early in the time course of the epidemic, few historic controls may be available and extrapolation to previous outbreaks may be unsound.

Response-adaptive randomization designs may be favourable because they minimize patients allocated to ineffective treatments, and so may be preferable from a patient and ethical perspective. In these designs no type I error inflation because of changing treatment effects over time compared to the complete randomization Bayesian design was observed.

A key aim of all designs is to reduce the size of or eliminate the control group because of ethical and operational constraints. Relying on historic information is problematic when the historic data contradicts current data, e.g. when the CFR changes over time. Robust methods for constructing priors incorporating historic information have been proposed (e.g. [26, 27]). The operating characteristics of designs using such priors constructed from historic information across several outbreaks, are a topic of future research. An alternative which also uses historic information is the MSA design of [15], which starts with a single-arm but which can switch to a two-arm randomized controlled design if the absolute treatment effect is found to not be large enough.

The comparison of the clinical trial designs is based on a selected number of simulation scenarios informed by epidemiologic data from the 2013-2016 West Africa EVD outbreak. Future outbreaks of EVD or of different infectious diseases will have different characteristics which might result in different conclusions about the appropriate clinical trial designs.

This work advances one approach to enabling clinical researchers conducting trials in challenging circumstances to best serve their patients, that is, by harnessing routinely collected epidemiological information to identify trial designs most likely to succeed at a particular point during an outbreak. Further extrapolation of this work to outbreaks with different transmission patterns will be helpful, given the continued risk of emerging infectious disease outbreak, including pandemic influenza.

## Supporting information

S1 Appendix Cumulative incidence function. (PDF)

S2 Appendix Spline model. (PDF)

S3 Appendix General flowchart for all designs. (PDF)

S1 Table Stopping boundaries and cumulative sample sizes at each analysis for the frequentist designs. (PDF)
