# Supplementary material for: Performance of different clinical trial designs to evaluate treatments during an epidemic
Source: PLoS One. 2018 Sep 11;13(9):e0203387. doi: 10.1371/journal.pone.0203387 (PMC6133355; doi:10.1371/journal.pone.0203387)
Supplement: S1 Appendix — (PDF) [file pone.0203387.s001.pdf]

S1 Appendix Cumulative incidence function.

Define the all-cause survival function at time t by

$$S(t) = \exp(-\int_0^t \{h_R(s) + h_D(s)\}ds),$$

i.e. the probability that neither event occurs until time t. For cause-specific hazards of recovery h_R and death h_D the cumulative incidence function at time t is given by

$$\text{CIF}(t) = \int_0^t h_D(s)S(s)ds.$$

The case fatality rate at Day 14 is then simply $p = \text{CIF}(14)$. A non-parametric estimator of the CIF is given by

$$\widehat{\text{CIF}}(t) = \int_0^t \hat{S}(s)d\hat{\Lambda}_D(s),$$

where $\hat{S}$ is the Kaplan-Meier estimator of S and $\hat{\Lambda}_D$ is the Nelson-Aalen estimators of the cause-specific hazard of death. We have used the R package *etm* [25] to calculate the estimator.
