# Supplementary material for: Performance of different clinical trial designs to evaluate treatments during an epidemic
Source: PLoS One. 2018 Sep 11;13(9):e0203387. doi: 10.1371/journal.pone.0203387 (PMC6133355; doi:10.1371/journal.pone.0203387)
Supplement: S2 Appendix — (PDF) [file pone.0203387.s002.pdf]

S2 Appendix Spline model. The log-log transformation of the survival function is modelled as a natural cubic spline function of $\log(t)$ [13, 14].

$$\log(-\log(S(t))) = \gamma_0 + \gamma_1 \log(t) + \gamma_2 v_1(\log(t)) + \ldots + \gamma_{m+1} v_m(\log(t))$$

where $v_j(x)$ is the j-th basis function

$$v_j(x) = (x - k_j)_+^3 - \lambda_j (x - k_{min})_+^3 - (1 - \lambda_j)(x - k_{max})_+^3,$$

where $\lambda_j = (k_{max} - k_j)/(k_{max} - k_{min})$ and $(x - a)_+ = \max(0, x - a)$.

Two separate spline models with 4 internal knots are fitted for the two cause-specific hazards for recovery and death based on the complete data of all confirmed and hospitalized cases. The location of the knots and the values of γ_j are given in Table A.

Table A: **Spline models.**

		**Index j**					
**Model**	**Parameter**	1	2	3	4	5	6
1	k_j	-1.39	2.57	2.77	3.00	3.22	5.38
	γ_j	-7.85	0.67	-1.94	-6.47	16.67	-8.27
2	k_j	-1.39	1.79	2.08	2.40	2.77	5.57
	γ_j	-5.52	0.97	-1.56	0.34	2.81	-1.63

Knots (k_j) on the log-scale and coefficients (γ_j) of the two spline models ($j = 1, \ldots, 6$).
