# Supplementary material for: Performance of different clinical trial designs to evaluate treatments during an epidemic
Source: PLoS One. 2018 Sep 11;13(9):e0203387. doi: 10.1371/journal.pone.0203387 (PMC6133355; doi:10.1371/journal.pone.0203387)
Supplement: S3 Appendix — (PDF) [file pone.0203387.s003.pdf]

S3 Appendix General flowchart for all designs.

All of the designs can be illustrated in a single flowchart (Fig A). In the two-arm single-stage designs $K = 1$ and $J = 1$. In the multi-arm designs in our simulations $K = 5$ and $J = 4$. For the frequentist designs the efficacy and futility measures are always equal to the standardized test statistic, i.e. $e_{kj} = f_{kj} = Z_{kj}$ and the efficacy and futility thresholds are the upper and lower stopping boundaries with $a_K = b_K$ in order to force a decision at the final analysis.

For the Bayesian designs the efficacy measure is the probability $e_{kj} = P(p_j < p_0|\text{Data}_k)$ given the data Data_k at the k-th analysis. The futility measure is $f_{kj} = P(p_j < p_0 + 0.1|\text{Data}_k)$. The efficacy and futility thresholds are $a_k = 0.1$ and $b_k = 0.99$ $(k = 1, \dots, K)$.

For the designs with complete randomization the allocation probabilities are $q_j = 1/(\tilde{K} + 1)$ for all $j = 1, \dots, J$, where $\tilde{K}$ is the number of remaining treatment arms at stage k. For the response-adaptive randomization designs the allocation probabilities are initially $q_j = 1/(K + 1)$ and are then updated at the interim analysis according to Eq (2).

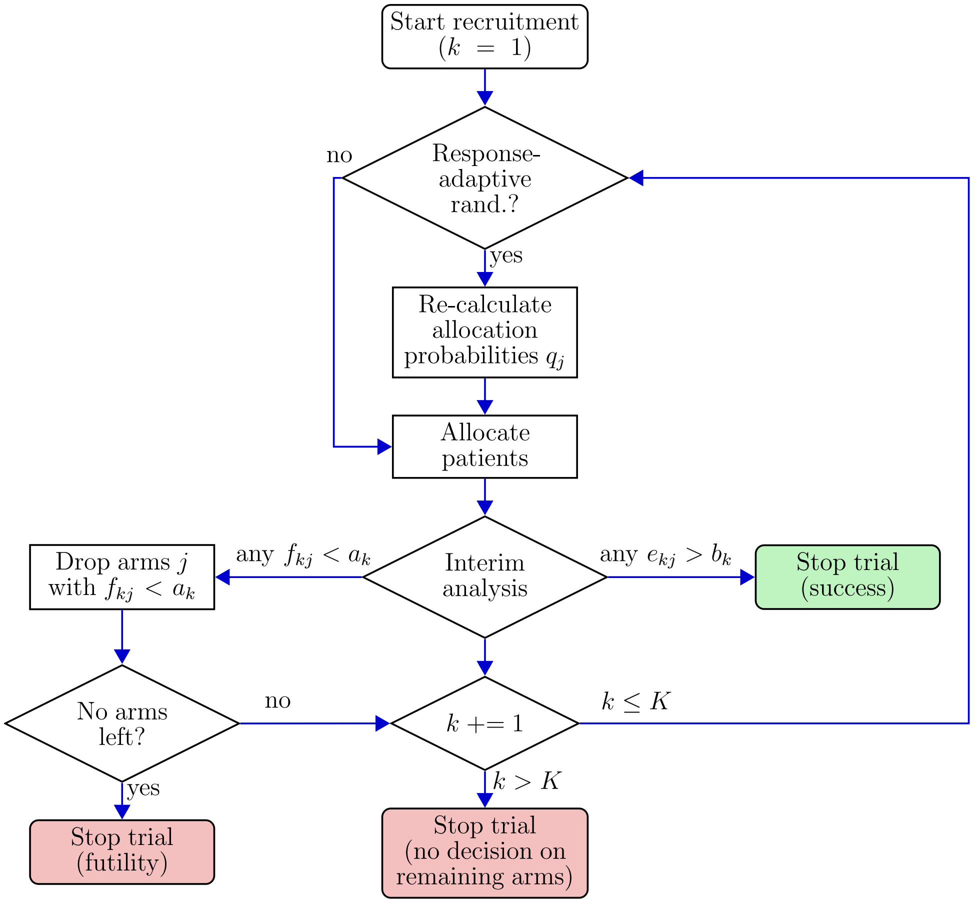

Figure A: **General flowchart for all designs.** At each of the at most K (interim) analyses the design-specific efficacy measures e_{kj} and futility measures f_{kj} $(k = 1, \dots, K; j = 1, \dots, J)$ for all remaining treatment arms are compared to efficacy thresholds b_k and futility thresholds a_k. Patients are allocated according to allocation probabilities q_{kj}.
