# Supplementary material for: Performance of different clinical trial designs to evaluate treatments during an epidemic
Source: PLoS One. 2018 Sep 11;13(9):e0203387. doi: 10.1371/journal.pone.0203387 (PMC6133355; doi:10.1371/journal.pone.0203387)
Supplement: S1 Table — (PDF) [file pone.0203387.s004.pdf]

S1 Table Stopping boundaries and cumulative sample sizes at each analysis for the frequentist designs.

Table A: **Stopping boundaries and cumulative sample sizes at each analysis for the frequentist designs.**

			**Stopping boundary**	
**Design**	**Analysis**	**Sample size per arm**	Lower	Upper
TACC	1	105	1.96	1.96
GSD	1	22	-0.92	3.25
	2	44	-0.07	2.99
	3	66	0.66	2.69
	4	88	1.32	2.37
	5	110	1.97	1.97
MAMS	1	22	-0.67	5.15
	2	44	-0.41	3.72
	3	66	-0.04	3.12
	4	88	0.33	2.76
	5	110	2.54	2.54
